# Speech Perception Benefits of Internet Versus Conventional Telephony for Hearing-Impaired Individuals

**DOI:** 10.2196/jmir.1818

**Published:** 2012-07-16

**Authors:** Georgios Mantokoudis, Patrick Dubach, Flurin Pfiffner, Martin Kompis, Marco Caversaccio, Pascal Senn

**Affiliations:** ^1^Cochlear Implant DivisionDepartment of Otorhinolaryngology, Head & Neck Surgery, InselspitalUniversity of BernBernSwitzerland

**Keywords:** VoIP, Internet telephony, hearing impaired, communication

## Abstract

**Background:**

Telephone communication is a challenge for many hearing-impaired individuals. One important technical reason for this difficulty is the restricted frequency range (0.3–3.4 kHz) of conventional landline telephones. Internet telephony (voice over Internet protocol [VoIP]) is transmitted with a larger frequency range (0.1–8 kHz) and therefore includes more frequencies relevant to speech perception. According to a recently published, laboratory-based study, the theoretical advantage of ideal VoIP conditions over conventional telephone quality has translated into improved speech perception by hearing-impaired individuals. However, the speech perception benefits of nonideal VoIP network conditions, which may occur in daily life, have not been explored. VoIP use cannot be recommended to hearing-impaired individuals before its potential under more realistic conditions has been examined.

**Objective:**

To compare realistic VoIP network conditions, under which digital data packets may be lost, with ideal conventional telephone quality with respect to their impact on speech perception by hearing-impaired individuals.

**Methods:**

We assessed speech perception using standardized test material presented under simulated VoIP conditions with increasing digital data packet loss (from 0% to 20%) and compared with simulated ideal conventional telephone quality. We monaurally tested 10 adult users of cochlear implants, 10 adult users of hearing aids, and 10 normal-hearing adults in the free sound field, both in quiet and with background noise.

**Results:**

Across all participant groups, mean speech perception scores using VoIP with 0%, 5%, and 10% packet loss were 15.2% (range 0%–53%), 10.6% (4%–46%), and 8.8% (7%–33%) higher, respectively, than with ideal conventional telephone quality. Speech perception did not differ between VoIP with 20% packet loss and conventional telephone quality. The maximum benefits were observed under ideal VoIP conditions without packet loss and were 36% (*P *= .001) for cochlear implant users, 18% (*P *= .002) for hearing aid users, and 53% (*P *= .001) for normal-hearing adults. With a packet loss of 10%, the maximum benefits were 30% (*P *= .002) for cochlear implant users, 6% (*P *= .38) for hearing aid users, and 33% (*P *= .002) for normal-hearing adults.

**Conclusions:**

VoIP offers a speech perception benefit over conventional telephone quality, even when mild or moderate packet loss scenarios are created in the laboratory. VoIP, therefore, has the potential to significantly improve telecommunication abilities for the large community of hearing-impaired individuals.

## Introduction

Engaging in telephone conversations is a challenge for many hearing-impaired individuals, including hearing aid and cochlear implant users [1-4]. Reduced social connectivity likely mediates the well-known associations between hearing loss and depression, cognitive decline, reduced quality of life, and increased morbidity and mortality [5-8]. Any improvement in telecommunication would therefore be of great importance and may affect millions of people, as hearing loss is a common disease in modern societies: it affects 1 to 4 of every 1000 people born [9], approximately 16% of adults aged 20 to 69 years [10], and more than 80% of older adults aged 80 to 92 years [11].

The two main technical limitations of conventional telephones for hearing-impaired individuals are, first, the frequency restriction and second, the digital data compression used in conventional telephony to maximize the network infrastructure utilization.

In addition, issues related to coupling the telephone to a hearing aid or a cochlear implant may further reduce speech perception by the hearing-impaired end user, even when assistive telephone listening devices are used [12-14]. Finally, hearing-impaired individuals face additional intelligibility problems when the caller or receiver is surrounded by environmental noise.

From a theoretical perspective, Internet telephony (voice over Internet protocol [VoIP]) should offer improved speech perception to the end user, as the transmitted frequency range is double that of conventional telephones (0.1–8 kHz vs 0.3–3.4 kHz). The association between improved speech perception and the presentation of speech at higher bandwidths has been repeatedly shown in the literature [15-17]. Additionally, modernized versions of signal processing strategies for coding and decoding speech, so-called codecs, have recently been implemented into VoIP software with the aim of improving intelligibility, particularly under adverse network conditions.

**Figure 1:**
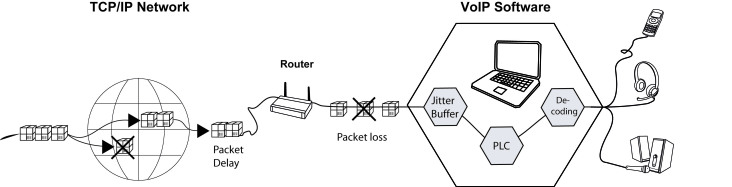
Each data packet originating in a voice over Internet protocol (VoIP)-sending device (not shown) takes a different route through the Internet (TCP/IP network) before arriving at the receiver. Data packets may be delayed or lost on the way. The VoIP software includes two solutions to improve audio quality in these cases: the jitter buffer collects the maximum number of data packets by waiting as long as needed and keeping the time delay to a minimum; and packet loss concealment (PLC) aims to reconstruct lost data packets. Finally, digital data packets are decoded and delivered to a VoIP-compatible user interface, such as a VoIP handheld telephone, a headset, or external loudspeakers.

These technical advantages of Internet telephony translate into improved speech perception by hearing-impaired and normal-hearing adults, at least when simulated under ideal laboratory conditions [18]. However, ideal network conditions are not constantly present in the World Wide Web, especially when Internet lines are overloaded [19]. Under adverse conditions, digital data packets may be delayed or lost. Lost data packets are usually described as a percentage of the original number of data packets sent through the network [20]. Despite software solutions designed to recover lost data packets (known as packet loss concealment; Figure 1), sound quality and speech perception may be negatively affected. For hearing-impaired individuals, even small differences in audio quality may have an important impact on speech perception. However, to date, no reports have addressed the relationship between packet loss in Internet telephony and speech perception by hearing-impaired individuals. To fill this gap, we assessed speech perception using standardized speech test material presented at a variety of simulated adverse VoIP qualities and compared it with speech perception under ideal conventional telephony conditions.

## Methods

We conducted all tests between January and June 2009 at the University Department of Otorhinolaryngology, Head and Neck Surgery, Inselspital, Bern, Switzerland. The study protocol was approved by the local institutional review board. All patients gave written informed consent.

### Test Participants

**Figure 2:**
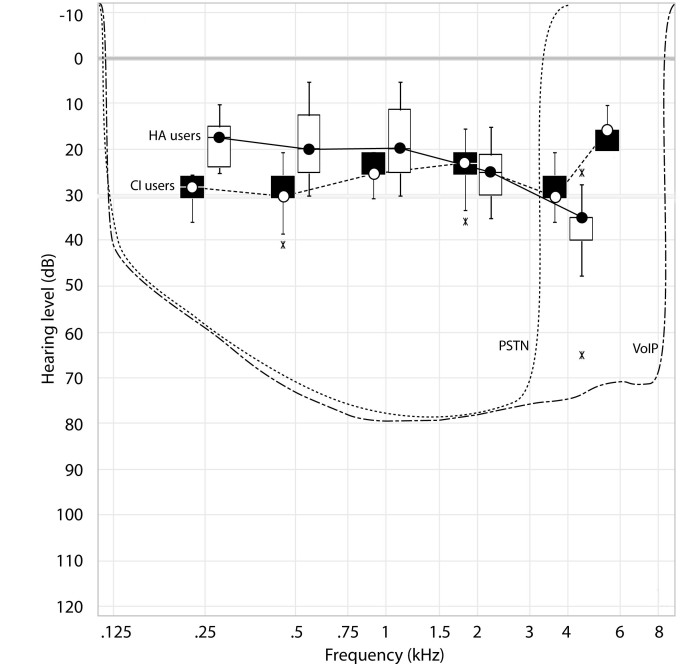
Lower quartile, median, upper quartile, and 1.5*interquartile range (X = outliers) of aided hearing thresholds in the free sound field for cochlear implant (CI) and hearing aid (HA) users. The analog telephone (public switched telephone network [PSTN]) speech signals are shown in dB hearing level as dotted lines. VoIP = voice over Internet protocol.

Participants in the study were 20 hearing-impaired adults, consisting of 10 users of cochlear implants and 10 users of hearing aids, and 10 normal-hearing adults. All test participants were at least 18 years old and were selected from the institution’s clinical database. Mean age was 46 years in the cochlear implant group, 68 years in the hearing-impaired group, and 35 years in the normal-hearing group. A total of 90% of participants who were tested in our previous experimental study [18] consented to participate in the present study and came to the laboratory for a second series of independent experiments. The same inclusion criteria used in the former study were applied; these were based on pure-tone audiograms, speech audiometry performance (the German Freiburger monosyllable test), and technical device specifications. Eligible cochlear implant users had processors that allowed stimulation beyond 5 kHz and aided minimum monosyllabic word discrimination scores of 50% or more at a 60-dB sound pressure level (Table 1). Eligible hearing aid users had moderate bilateral hearing loss with unaided sloping pure-tone audiometry thresholds, aided pure-tone audiometry with hearing gains beyond 3.4 kHz, and aided minimum monosyllabic word discrimination scores of 15% and 70% at sound pressure levels of 60 and 75 dB, respectively. Table 1 shows the aided speech discrimination scores for each individual and Figure 2 shows the aided hearing thresholds in free field, which reflects the degree of compensation for their hearing impairments and their ability to hear the telephone speech signal. The pure-tone audiometry thresholds and monosyllabic word discrimination scores of the normal-hearing control participants were within normal limits (100%).

**Table 1:** Clinical data for cochlear implant users and hearing aid users.

Participant		Sex	Age (years)	Hearing loss etiology	Device brand and model	Aided German monosyllable word discrimination score (%)			
						Ear	60 dB	75 dB	80 dB
**Cochlear implant users**									
	1	M^a^	77	Progressive	MED-EL Pulsar/Opus 2	Left	77.5	NA^b^	97.5
	2	M	17	Postmeningitic	MED-EL Pulsar/Opus 2	Left	77.5	NA	87.5
	3	F^c^	39	Congenital	MED-EL Pulsar/Opus 2	Right	62.5	NA	72.5
	4	F	69	Progressive	MED-EL C40+ Tempo+	Left	72.5	NA	85
	5	F	48	SHL^d^	MED-EL C40+ Tempo+	Right	77.5	NA	77.5
	6	F	61	Progressive	MED-EL Pulsar/Opus 2	Left	50	NA	65
	7	F	22	SHL	MED-EL C40+ Tempo+	Right	55	NA	75
	8	M	50	Congenital	MED-EL C40C Tempo+	Right	85	NA	80
	9	M	58	Meningitis	MED-EL C40C Tempo+	Left	70	NA	65
	10	F	23	Progressive	MED-EL C40C Tempo+	Right	55	NA	52.5
**Hearing aid users**									
	1	F	66	Presbycusis	BTE/Oticon Tego Pro VC	Left	100	100	NA
	2	M	77	Presbycusis	ITE/Bernafon Symbio XT	Left	100	100	NA
	3	M	86	Presbycusis	BTE/Phonak Piconet 2	Left	90	95	NA
	4	F	79	Presbycusis	ITE/Bernafon Neo 315	Left	75	95	NA
	5	M	91	Presbycusis	BTE/Widex Inteo	Left	25	85	NA
	6	M	62	Presbycusis	BTE/Phonak Una M AZ	Right	30	80	NA
	7	M	76	Progressive	BTE/Phonak Extra	Left	70	85	NA
	8	F	36	Congenital	BTE/GN ReSound Air	Right	100	100	NA
	9	M	63	Progressive	BTE/Phonak micro eXtra	Left	90	100	NA
	10	M	41	Progressive	BTE/Phonak Audéo	Right	70	95	NA

^a ^Male.

^b ^Not applicable.

^c ^Female.

^d ^Sudden hearing loss.

### Speech Perception Test Protocol

All tests were performed in the free sound field in a sound-treated room. The speech test material was played on an audio compact disc (CD) player connected to an audiometer (GSI 61; Grason-Stadler, Milford, NH, USA) and was reproduced using a pair of loudspeakers (Type 1030 A; Genelec Oy, Iisalmi, Finland) situated 1 m from the front of the patient’s head.

All tests were conducted monaurally. The most suitable ear was selected based on the inclusion criteria; if both ears were equally suitable, the ear commonly used for telephony was used. The opposite ear canal was occluded with an earplug (E.A.R. Classic, Aearo Technologies, Stockport, UK). The specified average attenuation of these earplugs is 24.6–41.6 dB in the 250- to 4000-Hz range. Bilateral cochlear implant users had to switch off one of their devices to produce homogeneous and comparable data. Monaural testing was necessary because not all individuals had the same degree of hearing loss on both sides, and speech perception performance varied between the first and second listening device. In addition, monaural testing more realistically reflects the use of a conventional telephone handset.

We used the standardized German Hochmair-Schulz-Moser (HSM) Sentence Test [21] in quiet and competing noise to test speech perception. This test consists of 30 lists with 20 sentences and 106 words per list to avoid learning effects. The sentences were presented in 4 different VoIP qualities (with 0%, 5%, 10%, and 20% packet loss) and ideal conventional telephone quality (Table 2) in quiet and with competing noise with 4 different signal to noise ratios (SNRs): 15, 10, 5, and 0 dB. We set the defined broadband noise signal to a constant level of 70 dB sound pressure level and changed the speech signal according to the SNR required by the standardized test protocol (range 70–85 dB sound pressure level). The audibility of the speech signal in relation to the aided hearing threshold is shown in Figure 2 for VoIP and analog telephone signals. Participants had to orally repeat the presented sentences, and the percentage of correctly understood words was assessed and used for comparison across conditions. A random permutation of all individuals was performed in conjunction with test order randomization to avoid order effects and selection bias. The participants were unaware of which condition was being tested at any time of the assessment.

**Table 2:** Frequency and filter characteristics for each audio quality

Codec	Frequency range (kHz)	Sampling rate	Fp (Hz)^a^	Fs (Hz)^b^	Ap (dB)^c^	As (dB)^d^
PSTN^e ^codec G.711	0.1–3.4	8 kHz G.711 A-Law	3900	4400	1	60
iPCMwb codec	0.1–8	16 kHz PCM^f^	8000	8500	1	60
CD low-pass filtered	0.1–8	16 kHz PCM	8000	8500	1	60

^a ^Frequency at the edge of the pass band.

^b ^Frequency at the beginning of the stop band.

^c ^Amount of ripple allowed in the pass-band (also called Apass).

^d ^Stop-band attenuation.

^e ^Public switched telephone network.

^f ^Pulse code modulation.

### Digital Generation of VoIP Audio Signals

The original audio CD files of the HSM Sentence Test were converted into a wave-format audio file using the Switch Audio File Converter software, version 1.05 (NCH Software Pty Ltd, Canberra, ACT, Australia). The speech and noise channels were mixed to mono wave files with a sampling rate of 44.1 kHz, thereby allowing identical signal processing for speech and noise. Before encoding, the audio files were low-pass filtered using MATLAB software, version 7.9.0.529 (The MathWorks, Inc, Natick, MA, USA).

The audio files were then converted again into raw files using Switch Audio File Converter software. To generate a VoIP simulation with different extents of packet loss, the raw data were encoded using simulation software (a voice engine demonstration application) in conjunction with a modern iPCMwb codec (0.1–8 kHz; Global IP Solutions, San Francisco, CA, USA). Table 2 shows the filter parameters. The public switched telephone network (PSTN) transmission has an upper limit of 3.4 kHz. In contrast to this, the frequency transmission of VoIP extends to 8 kHz, thereby preserving the high-frequency content of speech.

**Table 3:** Parameters of the voice over Internet protocol (VoIP) simulations.

Condition	Description (packet loss)	Loss rate (p)	Loss length (packets)	BurstR	Frame length (bytes)	q
0	Perfect	0.0	1.0			
1	Mild loss (5%)	0.05	1.15	1.1	640	0.87
2	Medium loss (10%)	0.10	1.30	1.2	640	0.77
3	Severe loss (20%)	0.20	2.0	1.43	640	0.5

We simulated 4 different scenarios: 1 scenario without packet loss and 3 scenarios with packet losses of 5%, 10%, and 20%. In Table 3, the parameter p represents the probability of packet loss. The average loss length defines the number of lost packets. The parameter BurstR is a measure of packet loss “burstiness,” as defined by Raake [22]. BurstR = 1.0 indicates that there was no correlation across packet losses, which were all independent and identically distributed with probability p (obtained by omitting the loss length parameter from the command line). BurstR >1.0 means that the packet losses tended to come in bursts; the larger the BurstR, the longer the bursts of losses. The variable BurstR is dimensionless. The parameter q is simply a translation of loss length into the transition probability from a *lost *to a *found *state of the underlying Markov model: q = 1 / loss length [23,24]. Finally, the encoded data sizes for a frame, the frame length or payload, are indicated in bytes.

### Digital Generation of PSTN Audio Signals

To simulate conventional telephone audio quality, we implemented a PSTN G.711 A-Law codec, which is a standard in PSTNs, in the Switch Audio File Converter software*. *We coded the files at a sampling rate of 8 kHz. Before encoding, we used MATLAB software to limit the upper frequency range of the original wave files to 4 kHz.

All 5 audio CDs (4 VoIP simulations and 1 PSTN quality simulation) were reproduced using an active loudspeaker system (Genelec Type 1030 A). They were calibrated in the free sound field using a Type 2636 measuring amplifier and a Type 4133 FF measuring microphone connected to a Type 2619 preamplifier (all from Brüel & Kjær Sound & Vibration Measurement A/S, Nærum, Denmark). We used a Pistonphone Brüel & Kjær 4288 precision calibrator to calibrate the measurement arrangement. The final measurements showed no difference in the sound pressure levels of speech signals across different audio signals.

### Statistical Analysis

W used a 2-tailed Wilcoxon matched-pairs signed rank test to compare the scores obtained under different VoIP versus telephone quality simulations. For the condition with no packet loss (condition 0, Table 3), a 1-tailed test was applied because of the expected superiority of VoIP under this condition [18,25]. *P *< .05 was considered significant. When applying a Bonferroni correction for multiple testing, *P*_Bonf _£ .0125 was considered significant.

## Results

**Figure 3:**
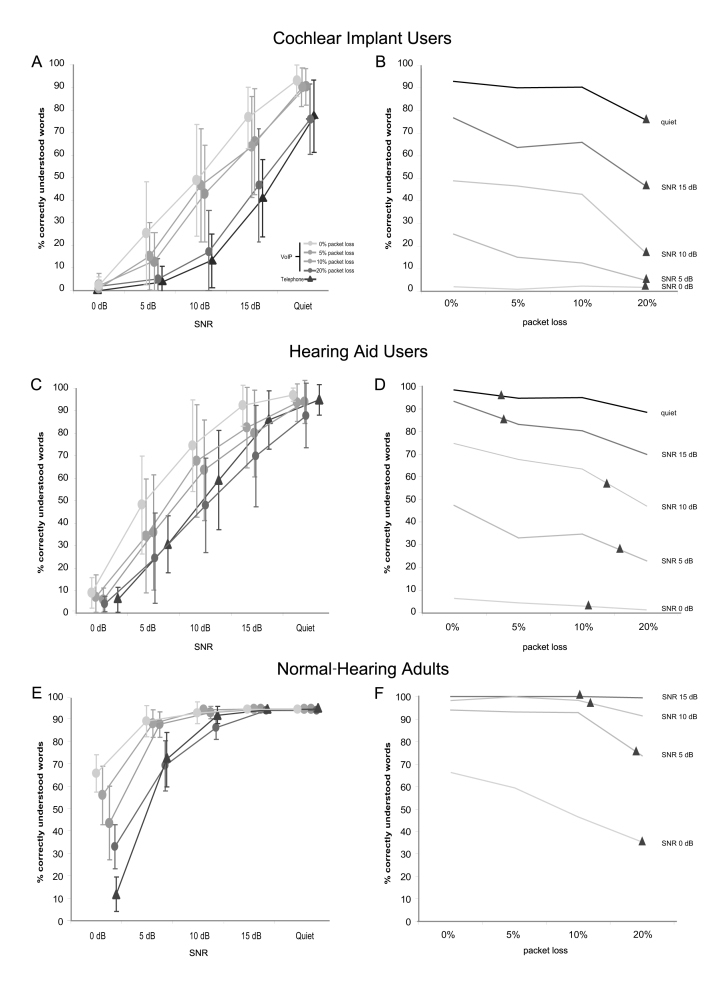
Speech perception scores assessed with the HSM Sentence Test are plotted against different signal to noise ratios (SNRs) for cochlear implant users (A), hearing aid users (C), and normal-hearing adults (E) for 4 different VoIP qualities (0%, 5%, 10%, and 20% packet loss) and 1 ideal conventional telephone quality. The impact of different network conditions with increasing data packet loss (x-axis) on word discrimination scores is shown for different SNRs in B, D, and F. The black triangle indicates the speech perception level corresponding to a conventional telephone with a constant and stable transmission. VoIP = voice over Internet protocol.

Across all test groups, speech perception scores assessed with different VoIP qualities versus conventional telephone quality were higher in 39 out of 60 test conditions (Figure 3 and Table 4). The average advantage of VoIP in the 39 conditions was 14.6% (range 1%–53%), and the differences were statistically significant in 23 conditions (*P *< .05). Mean speech perception scores were 15.2% (range 0%–53%) higher in the sentence test using VoIP with no packet loss, 10.6% (4%–46%) with mild packet loss, and 8.8% (7%–33%) with medium packet loss across the 3 groups (Table 4). Scores obtained under VoIP conditions with severe packet loss were similar to those obtained with conventional telephone quality.

Cochlear implant users showed improved speech perception using VoIP in 19 out of 20 test conditions (Figure 3, part A). On average, speech perception scores were 15.3% (range 1%–36%) higher with VoIP. The differences reached statistical significance in 13 conditions (Table 4). In only 1 condition with severe packet loss did we find a disadvantage for VoIP versus conventional telephone quality; this 1% difference was not statistically significant.

Hearing aid users had improved speech perception scores with different VoIP qualities in half of the test conditions (Figure 3, part C). The mean advantage was 7.1% (range 2%–18%); this advantage was statistically significant in 3 test conditions with no packet loss (Table 4). In 9 other test conditions with increased packet loss, speech perception scores were on average 6.2% (1%–17%) lower with VoIP. The negative differences were statistically significant under 4 conditions with severe packet loss (Table 4).

Normal-hearing adults showed an average benefit of 20.8% (range 1%–53%) with different VoIP qualities under half of the conditions (Figure 3, part E). Under 7 conditions, the differences were statistically significant. Under 6 conditions with medium and severe packet loss, an average disadvantage of 2.2% (1%–6%) was found for VoIP; one of these conditions showed a statistically significant disadvantage (Table 4).

When we experimentally increased VoIP packet loss from 0% to 10%, speech perception scores dropped only mildly (Figure 3, parts B, D, and F). When the packet loss increased to 20%, speech perception scores dropped more sharply. The scores achieved with VoIP qualities of 5% to 10% packet loss were typically still better than those obtained with simulations of ideal conventional telephone quality (represented by black triangles in Figure 3, parts B, D, and F).

**Table 4:** Mean differences in speech perception scores (D%) assessed with different voice over Internet protocol qualities (degree of packet loss) versus conventional telephone quality using the Hochmair-Schulz-Moser (HSM) Sentence Test.

Participant group	SNR (dB)^a^	Packet loss							
		None (0%)		Mild (5%)		Medium (10%)		Severe (20%)	
		D%	*P *value	D%	*P *value	D%	*P *value	D%	*P *value
CI^b ^users	0	+2	NA^c^	+1	NA	+2	NA	+2	NA
	5	+21	.002^d^	+11	.01^d^	+9	.008^d^	+1	.81
	10	+36	.001^d^	+34	.002^d^	+30	.002^d^	+4	.04^e^
	15	+36	.001^d^	+23	.01^e^	+25	.006^d^	+6	.25
	Quiet	+16	.001^d^	+16	.002^d^	+16	.002^d^	–1	.92
HA^f ^users	0	+4	.16	+2	.69	+0	>.99	–1	.02^e^
	5	+18	.002^d^	+4	.56	+6	.38	–6	.16
	10	+16	.003^d^	+9	.08	+4	.32	–12	.02^e^
	15	+6	.004^d^	–4	.65	–7	.25	–17	.004^d^
	Quiet	+2	.16	–1	.31	–1	.56	–7	.004^d^
NHA^g^	0	+53	.001^d^	+46	.002^d^	+33	.002^d^	+22	.002^d^
	5	+17	.003^d^	+16	.002^d^	+16	.004^d^	–3	.49
	10	+1	.21	+3	.06	+1	.47	–6	.009^d^
	15	0	NA	0	NA	–1	NA	–1	>.99
	Quiet	0	NA	0	NA	–1	NA	–1	NA

^a ^Signal to noise ratio.

^b ^Cochlear implant.

^c ^Not applicable.

^d ^Statistically significant with Bonferroni correction.

^e ^Statistically significant without Bonferroni correction.

^f ^Hearing aid.

^g ^Normal-hearing adults.

## Discussion

### Key Findings

The present study confirmed that simulations of Internet versus conventional telephony quality are associated with improved speech perception by hearing-impaired and normal-hearing adults under perfect network conditions without packet loss or delay in the laboratory. The advantage for cochlear implant users and normal-hearing adults persists, even when the VoIP quality is reduced by 5% and 10% packet loss. Similarly, hearing aid users also scored higher with VoIP than with conventional telephony, but the differences did not reach statistical significance.

In general, speech perception scores assessed with VoIP quality remained good until a packet loss of 10%. Interestingly, VoIP simulation under severely adverse network conditions (with a packet loss of 20%) was not inferior to a perfect conventional telephone simulation in the majority of test conditions and for most of the tested participants.

### Impact of Frequency Range, Coding Strategy, and SNR on Speech Perception

Our research group earlier showed the superiority of VoIP versus conventional telephony simulations under perfect network conditions [18]. The main reason for this advantage is the enlarged frequency range offered by VoIP (0.1–8 kHz vs 0.3–3.4 kHz). The association of improved speech perception with presentation of speech at higher bandwidths has been repeatedly shown [15-17].

Cochlear implant users had the lowest speech perception scores of all 3 tested groups in our study (Figure 3), which is explainable because they have the highest degree of hearing loss. Interestingly, cochlear implant users reached the highest gain when tested with VoIP versus conventional telephony. The main reason for this is probably technical. Cochlear implant devices transmit a broad frequency range (on average 0.2–7.9 kHz, as verified prior to testing; Figure 2). Therefore, cochlear implant users can fully exploit the enlarged frequency range offered by VoIP. In contrast, most hearing aids used by our test participants do not transmit up to 8 kHz (on average 0.2–6.7 kHz according to the manufacturer’s specifications). Hearing aid users can therefore not exploit the full frequency range offered by VoIP. The enlarged frequency range is audible to hearing aid users with functional thresholds below 40 dB at 4 and 6 kHz, respectively (Figure 2). They still benefit from the enlarged frequency range, but to a lesser extent.

The second reason for the advantage of VoIP is the conservation of high audio quality through digital signal processing using the chosen iPCMwb codec. In the first study by our group, speech perception scores that were assessed with VoIP quality were equal to scores obtained with frequency-restricted (0.1–8 kHz), uncompressed audio CD quality [18]. This means that through compression of digital data no relevant information for speech perception is lost. Additionally, modern VoIP codecs offer a constant full frequency range transmission even under adverse network conditions. The bit rate of the chosen iPCM-wb codec was variable (minimum at 36 kbit/s for silence and low levels) at an average rate of 80 kb/s and constant sampling rate of 16 kHz, which ensured a high-quality audio performance over heavily loaded packet networks.

Speech perception is more challenging with increasing competing noise, or decreasing SNRs. In particular, elderly hearing-impaired individuals with predominant high-frequency hearing loss suffer in noisy test conditions; in addition to complication from competing noise, the high-frequency content of speech is missing. VoIP may be helpful, because it transmits the high-frequency content of speech and because it offers the possibility of presenting the speech signal simultaneously to both ears through external loudspeakers, thereby allowing binaural hearing, which is a well-known advantage for speech perception in noise. Additionally, wired or wireless links enabling binaural hearing from 1 telephone signal are already available for hearing aids and cochlear implants.

### Packet Loss

The measurement of packet loss under real VoIP transmission is a challenge for many VoIP companies because there is no constant data transmission over the Internet [26]. Packet loss of speech data may have a significant impact on speech audio quality [27]. The network transmission of voice data packets depends on the Internet infrastructure and transmission capacity, both of which may vary across companies and countries [28]. The transmission capacity may be reduced when Internet lines are overcharged. This situation can occur during rush hours, for example.

A decade ago, when VoIP telephony was not so highly developed, the average packet loss for a large number of measurement traces has been reported to be below 8% (p < 0.08 and q > 0.8) [24]. Since then, the Internet infrastructure, VoIP-compatible devices, and VoIP software solutions have been drastically improved. In 2004, one author already postulated that the packet loss should be held lower than 1% to ensure excellent VoIP transmission [29]. Nowadays, this request seems to be met, at least for most of the highly industrialized countries.

It can therefore be assumed that telecommunication using VoIP should substantially improve speech perception compared with conventional telephony under real network conditions, since the benefit of VoIP was measurable for most of the test participants up to an experimental packet loss of 10% in our study. A packet loss of more than 10% has a significant impact on sound quality with tone bursts, interruptions, extended time delay, and jitter of the audio signal. This is shown in Figure 3, parts B, E, and H, with degradation of speech intelligibility beyond 10% packet loss.

The calculations and models of packet loss depend on the measuring method used [30-35]. However, passive or active real-time packet loss monitoring and measurements are still challenging for many researchers and network engineers.

To our knowledge, no other group has assessed the speech perception of hearing-impaired individuals using Internet telephony under adverse network conditions. The results of the present study therefore fill an important gap. Measuring packet loss under controlled laboratory conditions offers the opportunity to systematically address a highly variable phenomenon in the real network.

### Jitter Buffer, Time Delay, and Packet Loss Concealment

Many technical parameters that may further influence speech perception by the end user have not been addressed in the present study. Every conversation can be disturbed when data packets arrive late to the receiver [28]. A jitter buffer is a part of the software solution for this problem. It collects all relevant voice data packets by waiting as long as needed and minimizing the time delay (Figure 1). Packet losses can also be induced locally by setting a low playout delay in the jitter buffer. This may lead to bursts of packet loss and increased jitter, with further degradation of audio speech quality. Additionally, time delays and echo may affect speech perception; however, we did not test these factors. Nonetheless, experiences with VoIP and broadband access networks have shown only small packet delay variations with minimal delay and jitter [29]. Similarly, packet loss concealment algorithms (Figure 1) in the VoIP software reconstruct speech information when data packets are lost [28,29]. Because speech perception may vary with the use of different packet loss concealment settings and codecs, we cannot generalize our results for all VoIP software available on the market. VoIP audio quality may further be influenced by wireless network conditions [34].

### Practical Usefulness

Our test results may be important for hearing-impaired individuals, including hearing aid and cochlear implant users, because there is now strong experimental evidence for real improvement in speech perception when using VoIP instead of conventional telephones. The study is also important for physicians, audiologists, cochlear implant engineers, speech therapists, and other professionals who care for hearing-impaired individuals. Professionals should encourage hearing-impaired individuals to try VoIP, which is typically downloadable at no cost from most providers. Patients who already own a computer may be able to gain the benefits of VoIP at no cost. The use of external loudspeakers connected to the computer may further improve speech perception by permitting bilateral hearing and additional amplification through the volume control, which should be mentioned to the patients. Hearing aid and cochlear implant accessories, such as an FM transmitter and 3.5-mm audio jack, may also be helpful for coupling the computer directly to the hearing device. Patients should be advised that both sender and receiver should have a good microphone and loudspeaker system to take advantage of VoIP’s broadband advantage over conventional telephony.

### Conclusions

Speech perception by hearing-impaired individuals and normal-hearing adults is improved when using perfect VoIP versus perfect conventional telephony transmission under controlled laboratory conditions. The superiority of VoIP persists even under experimental adverse network conditions, but not to the same extent and not for all tested individuals. Cochlear implant users seem to benefit more than hearing aid users because their devices are better suited to exploit VoIP’s broadband frequency range.

## Abbreviations

CD: compact disc
HSM: Hochmair-Schulz-Moser
PSTN: public switched telephone network
SNR: signal to noise ratio
VoIP: voice over Internet protocol
